# The association between glycemic variability and diabetic cardiovascular autonomic neuropathy in patients with type 2 diabetes

**DOI:** 10.1186/s12933-015-0233-0

**Published:** 2015-06-04

**Authors:** Ji Eun Jun, Sang-Man Jin, Jongha Baek, Sewon Oh, Kyu Yeon Hur, Myung-Shik Lee, Moon-Kyu Lee, Jae Hyeon Kim

**Affiliations:** Division of Endocrinology and Metabolism, Department of Medicine, Samsung Medical Center, Sungkyunkwan University School of Medicine, 81 Irwon-Ro, Gangnam-Gu, Seoul, 135-710 Republic of Korea; Division of Endocrinology and Metabolism, Department of Internal Medicine, Gyeongsang National University Hospital, Gyeongsang National University School of Medicine, Jinju, Republic of Korea

**Keywords:** Glycemic variability, Cardiovascular autonomic neuropathy, Continuous glucose monitoring, Type 2 diabetes mellitus

## Abstract

**Background:**

It is presently unclear whether glycemic variability is associated with diabetic cardiovascular autonomic neuropathy (CAN). The aim of this study was to examine whether short- and/or long-term glycemic variability (GV) contribute to CAN.

**Methods:**

A total of 110 patients with type 2 diabetes who underwent three-day continuous glucose monitoring (CGM) completed five standardized autonomic neuropathy tests. Short-term GV was measured by the standard deviation (SD), coefficient of variation (CV) of glucose, and the mean amplitude of glycemic excursions (MAGE) in CGM. HbA1c variability was calculated from the intrapersonal SD, adjusted SD, and CV of serial HbA1c over 2-year period. CAN was defined as the presence of at least two abnormal parasympathetic function tests. The severity of CAN was evaluated by total scores of five autonomic function tests.

**Results:**

In univariate analysis, not only SD and CV in CGM but also all parameters of HbA1c variability were significantly higher in the patients with CAN (*n* = 47, 42.7 %) than in those without CAN. In multivariate analysis, CV (Odds ratio [OR] 1.07, 95 % confidence interval [CI] 1.01–1.13; *p* = 0.033), but neither SD nor MAGE in CGM, independently correlated with the presence of CAN. All parameters of HbA1c variability, such as SD of HbA1c (OR 12.10 [95 % CI 2.29–63.94], *p* = 0.003), adjusted SD of HbA1c (OR 17.02 [95 % CI 2.66–108.86], *p* = 0.003), and log CV of HbA1c (OR 24.00 [95 % CI 3.09–186.48], *p* = 0.002), were significantly associated with the presence of CAN. The patients with higher HbA1c variability had an increased risk of advanced CAN.

**Conclusion:**

CV in CGM and all parameters of HbA1c variability were independently associated with the presence of CAN in patients with inadequately controlled type 2 diabetes requiring CGM.

**Electronic supplementary material:**

The online version of this article (doi:10.1186/s12933-015-0233-0) contains supplementary material, which is available to authorized users.

## Background

Diabetic cardiovascular autonomic neuropathy (CAN) is one of several common diabetic microvascular complications. CAN involves autonomic nerve fibers innervating the heart and blood vessels, and consequentially represents a significant cause of cardiovascular morbidity and mortality in diabetic patients [1]. A growing body of clinical and laboratory evidence suggests that glycemic variability (GV) may play a role in developing autonomic neuropathy independently of chronic hyperglycemia, by contributing to oxidative stress that leads to neural damage [2, 3]. Nevertheless, there has been considerable debate over whether glycemic instability confers a risk of diabetic complications in addition to that predicted by mean glycemia alone [4].

Glycemic variability refers to short-term fluctuations in glycemia, such as within-day variability, variability between daily means, or within-series variability [5]. Early post-hoc analysis of data from the Diabetes Control and Complications Trial (DCCT) using seven-point self-monitoring blood glucose (SMBG) levels revealed no significant association between short-term GV and diabetic retinopathy, nephropathy [6], or neuropathy, which was defined as abnormal nerve conduction, sensory signs, and heart rate variability in type 1 diabetes [7]. The Epidemiology of Diabetes Interventions and Complications (EDIC) study, which was extended from the DCCT, also found no evidence of a contribution of short-term GV to retinopathy or nephropathy [8]. However, one of the limitations in those studies was that the seven-point glucose profiles did not adequately reflect overall glycemic patterns.

Continuous glucose monitoring (CGM) is now regarded as a more accurate method for the assessment of glycemic variability than is SMBG [9]. Several studies [10, 11] have in fact demonstrated that increased short-term GV was associated with diabetic microvascular complications, by using CGM data.

Whereas CGM measures short-term fluctuation of glycemia, HbA1c variability reflects glycemic fluctuation over longer periods of time, as HbA1c reflects glycemic control over 2–3 months [12]. Two large trials [13, 14] reported that duration of diabetes, not SD of HbA1c, was an independent risk factor for diabetic retinopathy, whereas a subcohort analysis from a Finnish Diabetic Nephropathy (FinnDiane) study [15] reported that higher HbA1c variability (CV of HbA1c) was associated with an increased need for laser treatment in patients with type 1 diabetes. Microalbuminuria or CKD stage was more concordantly related to HbA1c variability independent of mean HbA1c in patients with type 2 diabetes [13, 16, 17]. Diabetic nephropathy is known as the most sensitive complication to changes in HbA1c [18].

Because the majority of studies regarding the effect of GV on diabetic microvascular complications have focused on retinopathy or nephropathy, little is known whether GV is associated with diabetic autonomic neuropathy, and in particularly with CAN. Thus far, one cross-sectional study [19] showed that heart rate variability, one of the earliest indicators of CAN, significantly correlated with GV (SD of mean glucose, M-value) measured by CGM in patients with type 2 diabetes. An additional small study [20] showed that MAGE calculated from CGM data affected sympathovagal balance in 26 type 2 diabetic patients without overt autonomic neuropathy. However, we have found no previous study on the influence of HbA1c variability on CAN.

The aim of this study was therefore to determine whether short-term GV measured by three-day CGM or HbA1c variability is associated with the presence and severity of CAN.

## Methods

### Study subjects

Using electrical medical records, we created a clinical database of 655 consecutive adult (age ≥ 18 years) patients with type 2 diabetes who underwent CGM in the outpatients’ clinic of Samsung Medical Center in Seoul, Republic of Korea between 2009 and 2011.

**Fig. 1:**
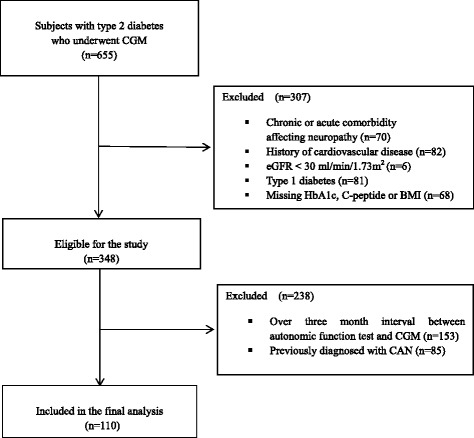
Selection of study subjects

Among these 655 patients, those with severe medical illness such as acute infection, liver cirrhosis, thyroid disease (either hypothyroidism or hyperthyroidism), or malignancy (*n* = 70); those with past medical history of cardiovascular disease such as myocardial infarction, stroke, coronary, carotid, or lower limb revascularization (*n* = 82), those with an eGFR calculated by the Chronic Kidney Disease Epidemiology Collaboration (CKD-EPI) formula of < 30 ml/min/1.73 m^2^ (*n* = 6); those with missing clinical data (*n* = 68); and those who were clinically diagnosed with type 1 diabetes (*n* = 81) were excluded from the study. The detailed characteristics of this cohort have been described elsewhere [21]. Among the remaining participants (*n* = 348), autonomic function tests were performed within three months of the date of CGM on 110 patients (72 males and 38 females) who had never been diagnosed with CAN (Fig. 1).

The baseline characteristics of the included patients, which were similar to the source cohort [21], are summarized in Table 1. The purposes for performing CGM in these patients were: unexplained large fluctuations in blood glucose values (*n* = 69), nocturnal hypoglycemia and/or hypoglycemia unawareness (*n* = 23), enrollment in clinical trial (*n* = 5), and adjustments in treatment regimen (*n* = 13).

**Table 1:** Demographic and clinical variables related to cardiovascular autonomic neuropathy

	Type 2 diabetes		
	No CAN (*n* = 63)	CAN (*n* = 47)	*p* value
Age (years)	59.5 ± 8.6	56.3 ± 8.1	0.055
Men/Women (%)	40/23 (64/36)	32/15 (68/32)	0.616
Body mass index (kg/m^2^)	25.7 ± 3.2	25.0 ± 2.9	0.225
Duration of diabetes (years)	11.7 ± 7.1	14.2 ± 7.2	0.076
Systolic blood pressure (mmHg)	126.9 ± 16.8	127.5 ± 15.1	0.851
Diastolic blood pressure (mmHg)	77.3 ± 10.5	77.1 ± 8.8	0.894
Lipid profile (mg/dL)			
Total cholesterol	155.7 ± 30.8	156.6 ± 33.6	0.885
Triglyceride	116.4 ± 62.0	134.1 ± 61.1	0.139
HDL-C	49.1 ± 11.3	46.3 ± 10.0	0.176
LDL-C	87.4 ± 23.8	90.5 ± 29.4	0.551
C-peptide (ng/mL)	2.3 ± 1.1	2.0 ± 1.1	0.108
eGFR (ml/min/1.73 m^2^)	83.5 ± 21.5	89.4 ± 19.3	0.142
Use of insulin, n (%)	16 (25)	25 (53)	0.003
Use of oral anti-diabetic drug, n (%)	55 (87)	40 (85)	0.740
Metformin, n (%)	48 (76)	39 (83)	0.386
Sulfonylurea, n (%)	24 (38)	16 (34)	0.662
Thiazolidinedione, n (%)	9 (14)	0 (0)	0.014
Glinide, n (%)	5 (8)	2 (4)	0.434
DPP 4 inhibitor, n (%)	22 (35)	17 (44)	0.892
α-glucosidase inhibitor, n (%)	15 (24)	9 (19)	0.558
Use of lipid-lowering agents, n (%)	46 (73)	36 (77)	0.670
Use of anti-hypertensive therapy, n (%)	47 (75)	32 (68)	0.452
ACE inhibitor or ARB, n (%)	51 (68)	25 (71)	0.717
CCB, n (%)	17 (27)	8 (17)	0.217
Thiazide, n (%)	6 (9.5)	6 (12.8)	0.589
Beta-blocker, n (%)	9 (14)	6 (12)	0.818
Use of aspirin, n (%)	32 (51)	22 (47)	0.679
Smoking (ex- or current smoker), n (%)	23 (37)	17 (36)	0.971

Data are mean ± SD, median (25th to 75th percentile) or percent

*CAN* cardiovascular autonomic neuropathy, *HDL-C* high density lipoprotein-cholesterol, *LDL-C* low density lipoprotein-cholesterol, *eGFR* estimated glomerular filtration rate, *DPP-4* dipeptidyl peptidase-4, *ARB* antiotensin receptor blocker, *CCB* calcium channel blocker

Clinical data (age, sex, body mass index [BMI], duration of diabetes in years, systolic blood pressure [SBP], diastolic blood pressure [DBP], insulin therapy, use of lipid-lowering agents and anti-hypertensive agents, smoking experience, HbA1c, eGFR, levels of high and low density lipoprotein–cholesterol [HDL and LDL], levels of triglyceride, and fasting C-peptide levels) were retrieved from electronic medical records on the first day of wearing the CGM device. All patients were informed of the purpose of the study and their consent was obtained. The protocol of this study was approved by the Institutional Review Board (IRB) of Samsung Medical Center.

### Assessment of glycemic variability

The parameters of short-term glycemic variability were obtained from CGM (Gold™ [Medtronic MiniMed, Northridge, CA, USA]) data. After being equipped with CGM devices, the enrolled subjects were monitored for 73.8 ± 15.0 consecutive hours each, averaging 885.4 ± 180.6 readings each during the monitoring period.

Short-term GV was assessed by measuring the standard deviation (SD) of all readings during the CGM, the overall glucose coefficient of variation (CV), and the mean amplitude of glycemic excursions (MAGE). CV (%) was calculated by dividing the SD by the mean of the corresponding glucose readings, and MAGE was automatically calculated using a computer program of the Diabetes Institute Karlsburg, applied exclusively to the middle 48 h of the CGM data [22].

HbA1c variability was evaluated using the intrapersonal SD and CV of serial measurements of HbA1c every three months during the 2-year period preceding recruitment, including HbA1c obtained on the first day of wearing the CGM device. It was undertaken a median of six times. In order to adjust for the effect of varying numbers of HbA1c measurements, we defined the adjusted SD of HbA1c as the SD of HbA1c divided by [n/(n–1)]^0.5^, where n is the number of HbA1c measurements [4, 23]. HbA1c levels were measured by high-performance liquid chromatography (HPLC), using a VARIANT II TURBO analyzer (Bio-Rad Laboratories, Hercules, CA, USA).

### Assessment of cardiovascular autonomic neuropathy

Patients were advised to avoid strenuous physical exercise, tobacco, and alcohol in the 24 h preceding the test, and to avoid coffee and eating for at least three hours prior to the test. Medications such as anti-histamines, anti-depressants, and β-blockers were withheld for 12 h prior to the test.

CAN was assessed by five standard cardiovascular reflex tests proposed by Ewing *et al.* [24]. Three of these measurements assess parasympathetic function: heart rate responses to deep breathing (exhalation: inhalation ratio), to standing (30: 15 ratio), and to the Valsalva maneuver (Valsalva ratio). The other two tests assess sympathetic function: blood pressure responses to standing and to a sustained handgrip. The heart rate responding to deep breathing, standing, and the Valsalva maneuver was assessed automatically from electrocardiography recordings using the DICAN evaluation system (Medicore Co., Ltd., Seoul, Korea).

Each sympathetic function test was graded as 0, each borderline test as 0.5, and each abnormal test as 1, while each parasympathetic function test was graded as 0, each abnormal test as 1 (Additional file 1: Table S1). Reference ranges of E:I ratio [25], valsalva ratio [26], and 30:15 ratio [27] varied across the age groups. Therefore, values below the lower limit of age-related reference range were considered abnormal (Additional file 1: Table S1). CAN was finally defined as the presence of at least two abnormal results among three parasympathetic tests [28].

The severity of CAN was quantified by the total CAN score, which summed the partial points obtained from each of the five autonomic function tests (minimum: 0, maximum: 5) [29].

### Definition of hypoglycemia

Hypoglycemia was defined as a blood glucose level of less than 70 mg/dL. Subgroup analysis was conducted in the patients who had over two episodes of hypoglycemia during middle 48 h of CGM.

### Statistical analysis

Normally distributed data was expressed as mean ± SD, whereas unevenly distributed data was presented as median (interquartile range: 25th to 75th percentile) for continuous variables, and ratios or percentages were used for categorical variables. Student’s *t*-test or the nonparametric Mann–Whitney *U*-test was used to compare the means of continuous variables. The categorical variables of the two groups were compared using the chi-square test.

Based on the outcome of univariate and colinearity analyses, multivariate binary logistic regression was performed to assess the independent association between GV and the presence of CAN. The covariates included in each multivariate model were age, sex, duration of diabetes, mean HbA1c [1, 30] and other known risk factors of CAN. The use of insulin treatment and each oral anti-diabetic medication was also included as a covariate, because it is a risk factor of hypoglycemia which could affect glycemic variability. Smoking and medications such as beta-blocker, ACE inhibitor/ARB, or aspirin, which could affect results of neuropathy function tests, were additionally adjusted. In addition, multivariate ordinary logistic regression was used to verify the association between GV and total CAN score.

Statistical analysis was executed using SAS version 9.3 (SAS Institute, Cary, NC). A value of *p* < 0.05 was considered statistically significant.

## Results

### Baseline characteristics of study subjects

A total of 110 subjects were classified into two groups according to the result of autonomic neuropathy tests: subjects with CAN (*n* = 47, 42.7 %) and subjects without CAN (*n* = 63, 57.3 %). Baseline characteristics of the two groups are summarized in Table 1.

The proportion of insulin user was significantly higher in CAN group. However, there was no statistical difference of age, diabetic duration, and fasting c-peptide level.

### The comparison of glycemic parameters between patients with and without CAN

CGM parameters except MAGE were significantly higher in CAN group. Mean HbA1c and all parameters of HbA1c variability were significantly higher in the CAN group as well (Table 2).

**Table 2:** The comparison of glycemic parameters between patients with and without cardiovascular autonomic neuropathy

	No CAN (*n* = 63)	CAN (*n* = 47)	*p* value
CGM parameters			
SD of glucose (mg/dL)	41.6 ± 15.0	51.7 ± 17.2	0.001
MAGE (mg/dL)	89.4 ± 37.1	103.1 ± 37.1	0.061
CV of glucose (%)	25.9 ± 7.9	30.1 ± 8.1	0.002
Mean glucose in CGM (mg/dL)	162.6 ± 47.4	172.2 ± 42.7	0.275
HbA1c variability over 2 years			
SD of HbA1c (%)	0.5 ± 0.3	0.8 ± 0.5	<0.001
Adjusted SD of HbA1c (%)	0.4 ± 0.2	0.7 ± 0.4	<0.001
CV of HbA1c	0.05 ± 0.04	0.09 ± 0.05	<0.001
Mean HbA1c over 2 years (%)	7.5 ± 1.0	8.4 ± 1.1	<0.001

Data are mean ± SD

Adjusted SD of HbA1c = SD of HbA1c/[n/(n–1)]^0.5^, where n is the number of HbA1c measurements

*CAN* cardiovascular autonomic neuropathy, *GV* glycemic variability, *CGM* continuous glucose monitoring, *MAGE* mean amplitude of glycemic excursions, *SD* standard deviation, *CV* Coefficient of variance

Since the hypoglycemia itself influence the results of CAN [31], we did additional subgroup analysis to the patients who developed recurrent hypoglycemia in CGM (*n* = 40). While only SD was significantly higher among CGM parameters, all parameters of HbA1c variability were significantly higher in CAN group with hypoglycemia events (Table 3).

**Table 3:** The comparison of parameters of glycemic variability between hypoglycemic patients with and without cardiovascular autonomic neuropathy

	Over two episodes of hypoglycemia in CGM data (*n* = 40)		
	No CAN (*n* = 29)	CAN (*n* = 11)	*p* value
CGM parameters			
SD in glucose (mg/dL)	42.0 ± 14.9	55.0 ± 19.6	0.029
MAGE (mg/dL)	94.5 ± 38.7	105.4 ± 37.7	0.424
CV in glucose (%)	30.5 ± 8.4	35.7 ± 7.2	0.074
Mean glucose in CGM (mg/dL)	137.1 ± 35.2	151.5 ± 38.1	0.264
HbA1c variability			
SD of HbA1c (%)	0.4 ± 0.2	0.6 ± 0.3	0.003
Adjusted SD of HbA1c (%)	0.4 ± 0.2	0.6 ± 0.2	0.003
CV of HbA1c	0.05 ± 0.02	0.08 ± 0.03	0.012
Mean HbA1c over 2 years (%)	7.3 ± 1.0	8.2 ± 1.0	0.019

Data are mean ± SD

*CAN* cardiovascular autonomic neuropathy, *CGM* continuous glucose monitoring, *MAGE* mean amplitude of glycemic excursions, *SD* standard deviation, *CV* coefficient of variance

### Binary logistic regression analysis for independent determinants of CAN

There was no significant relationship between confounding variables and the presence of CAN in the univariate analysis, except mean HbA1c (*p* < 0.001) and use of insulin (*p* = 0.003).

Separate multivariate binary logistic models were constructed for each parameter of GV in CGM and HbA1c variability, with the presence of CAN as a dependent variable (Table 4). Among CGM parameters, only CV of glucose (OR 1.07; CI. 1.01–1.13, *p* = 0.033) increased a risk of CAN in fully adjusted model (Model 3), whereas MAGE failed to demonstrate the same association in all models. Although SD of glucose (OR 1.04; CI. 1.01–1.17, *p* = 0.010) was associated with the presence of CAN after adjustment for age, sex, and duration of diabetes (Model 1), this association did not remain significant after additional adjustment of mean HbA1c (Model 2). Mean HbA1c remained significant in all multivariate models (OR 2.18 [1.26–3.77], *p* = 0.005; OR 1.98 [1.13–3.46], *p* = 0.033; OR 1.07 [1.01–1.13], *p* = 0.033 for each multivariate model including MAGE, SD, CV as a covariate, respectively).

**Table 4:** Binary logistic regression analysis for the associations between parameters of glycemic variability and the presence of CAN

Long-term GV	OR (95 % CI)	*p* value	Short-term GV	OR (95 % CI)	*p* value
SD of HbA1c			SD of glucose		
Crude	14.33 (3.45–59.48)	<0.001	Crude	1.04 (1.01–1.07)	0.002
Model 1	16.09 (3.55–73.00)	<0.001	Model 1	1.04 (1.01–1.07)	0.010
Model 2	9.31 (1.91–45.42)	0.028	Model 2	1.02 (0.99–1.05)	0.205
Model 3	12.10 (2.29–63.94)	0.003	Model 3	1.02 (0.99–1.05)	0.216
Adjusted SD of HbA1c			MAGE		
Crude	21.22 (4.24–106.30)	<0.001	Crude	1.01 (0.99–1.02)	0.064
Model 1	23.54 (4.28–129.69)	<0.001	Model 1	1.01 (0.99–1.02)	0.145
Model 2	13.39 (2.25–79.82)	0.004	Model 2	1.00 (0.99–1.02)	0.676
Model 3	17.02 (2.66–108.86)	0.003	Model 3	1.00 (0.99–1.02)	0.781
Log CV of HbA1c			CV of glucose		
Crude	19.90 (3.47–113.69)	0.001	Crude	1.07 (1.02–1.12)	0.009
Model 1	23.88 (3.77–151.52)	0.001	Model 1	1.07 (1.01–1.13)	0.014
Model 2	16.72 (2.43–115.04)	0.004	Model 2	1.06 (1.01–1.13)	0.028
Model 3	24.00 (3.09–186.48)	0.002	Model 3	1.07 (1.01–1.13)	0.033

Model 1 was adjusted for age, sex and duration of diabetes. Model 2 was additionally adjusted for mean HbA1c over 2 years. Model 3 was additionally adjusted for medication (insulin, oral anti-diabetic drug, aspirin, beta-blocker, ARB/ACE inhibitor) and smoking status

*CAN* cardiovascular autonomic neuropathy, *SD* standard deviation, *MAGE* mean amplitude of glycemic excursions, *CV* coefficient of variance

The parameters of HbA1c variability, such as SD of HbA1c (OR 12.10; CI. 2.29–63.94, *p* = 0.002), adjusted SD of HbA1c (OR 17.02; CI. 2.66–108.86, *p* = 0.003) and log transformed CV of HbA1c (OR 24.00; CI. 3.09–186.48 *p* = 0.002), were the independent risk factors of having CAN in fully adjusted models (Table 4, model 3). Mean HbA1c (OR 2.20 [1.28–3.78], *p* = 0.004) remained a significant covariate in the multivariate model constructed for the CV of HbA1c, but became insignificant in the models for SD or adjusted SD of HbA1c.

We did an additional analysis for SD of fasting glucose which was known as another indicator of long-term glycemic variability. SD of fasting glucose over 2 years (OR 1.02 [1.01–1.04], *p* = 0.025) was a significant risk factor of CAN in crude model. However, it lost its significance in multivariate logistic models after adjusted for mean HbA1c.

### The association between parameters of GV and the severity of CAN

We compared the effects of both short-term GV and HbA1c variability on total CAN score by an ordinary logistic regression analysis (Table 5). Total CAN score was categorized into 0–1.5 points (*n* = 62), 2–2.5 points (*n* = 33), and 3–5 points (*n* = 15). SD, CV of glucose in CGM, and all parameters of HbA1c variability were associated with increased odds of more advanced CAN in univariate analysis. However, only parameters of HbA1c variability such as SD of HbA1c (Odds ratio [OR] 15.40, 95 % confidence interval [CI] 4.56–52.01; *p* < 0.001), adjusted SD of HbA1c (OR 17.46 [95 % CI 3.86-79.06], *p* < 0.001), and log CV of HbA1c (OR 27.21 [95 % CI 4.28–172.91], *p* < 0.001) were associated with an increased odds of advanced CAN after adjusted for multiple confounding variables including mean HbA1c.

**Table 5:** Ordinary logistic regression analysis for the associations between parameters of glycemic variability and the severity of CAN

	CAN score category^†^			
	Univariate OR (95 % CI)	*p* value	Multivariate OR (95 % CI)	*p* value
Short-term GV				
SD of glucose	1.03 (1.01–1.06)	0.007	1.02 (0.99–1.05)	0.191
MAGE	1.01 (1.00–1.02)	0.059	1.01 (0.99–1.02)	0.398
CV of glucose	1.05 (1.00–1.10)	0.039	1.05 (1.00–1.10)	0.079
Long-term GV				
SD of HbA1c	13.79 (4.43–42.89)	<0.001	15.40 (4.56–52.01)	<0.001
Adjusted SD of HbA1c	18.83 (5.31–66.80)	<0.001	17.46 (3.86–79.06)	<0.001
Log CV of HbA1c	29.23 (5.47–156.11)	<0.001	27.21 (4.28–172.91)	<0.001

^†^CAN scores were categorized into 0–1.5 points (*n* = 62), 2–2.5 points (*n* = 33), and 3–5 points (*n* = 15)

Multivariate model was adjusted for age, sex, duration of diabetes, mean HbA1c, use of insulin, use of oral anti-diabetic drug, use of aspirin, use of beta-blocker, use of ARB/ACE inhibitor and smoking status

*CAN* cardiovascular autonomic neuropathy, *SD* standard deviation, *MAGE* mean amplitude of glycemic excursions, *CV* coefficient of variance

The relationship between each parameters of GV and total CAN score is illustrated by scatter plots in Additional file 1: Figure S1.

## Discussion

To the best of our knowledge, this is the first study to simultaneously evaluate the effects of both short-term GV in CGM and long-term GV represented by HbA1c variability on CAN. Our findings demonstrated that all parameters of HbA1c variability, and CV in CGM were independently associated with the presence of CAN.

In this study, CV in CGM, but neither SD nor MAGE in CGM, was associated with the presence of CAN when the mean glycemic level was adjusted. Among various CGM parameters of GV, parameters of relative GV such as CV was normalized by mean glucose levels, while parameters of absolute GV such as SD and MAGE were dependent of the mean glucose levels. CV was known to be a better method suited to exclude the influence of mean glucose than SD or MAGE [32]. It has been shown that only relative but not absolute parameters of GV predict hypoglycemia [33]. At least in part, the association between CV in CGM and the presence of CAN could be explained by that CAN itself is associated with a risk of hypoglycemia unawareness, which may result in greater GV in these patients. Likewise, an independent association between HbA1c variability and the presence of CAN was best demonstrated when CV of HbA1c was selected as a parameter of HbA1c variability. Inclusion of SD or adjusted SD of HbA1c in the multivariate model neutralized the effect of mean HbA1c, probably because the parameter SD of HbA1c, which was not normalized by the mean HbA1c, was closely associated with the mean HbA1c.

*In vitro* studies [34, 35] have shown that acute fluctuation of glycemia induced a greater triggering effect on oxidative stress than did chronic sustained hyperglycemia. Nevertheless, no direct link between acute fluctuation of glycemia and oxidative stress has been consistently reproduced in human studies [36, 37]. In the previous findings from the DCCT, in which there was no correlation between GV as measured by seven point SMBG and the incidence of microvascular complications during initial DCCT or four year follow-up period [6, 8]. In the DCCT, however, the degree of GV was measure by seven point SMBG, which provides only limited information on GV. Several studies using CGM indicated the association between GV measured by CGM and microvascular complication [10, 11, 38] and future lager study could provide more definite answers.

In this study, two-year variability of HbA1c was a strong correlate of CAN. This was consistent with the findings from the DCCT trial in which four-year SD of HbA1c had a marked association with the development of retinopathy and nephropathy [4]. The results of this study were also in accordance with a recent prospective study [16] in which the mean and SD calculated from three or four measurements of HbA1c over two years were sufficient to predict the progression of diabetic nephropathy in patients with type 2 diabetes. Moreover, previous literatures indicated both HbA1c and HbA1c variability were also independently associated with cardiovascular disease (CVD). Japanese prospective study [39] demonstrated that a higher level of HbA1c, even in non-diabetic range, was an independent risk factor for CVD, especially coronary heart disease and ischemic stroke. It is speculated that HbA1c threshold for diagnosing diabetes was lower than current diagnostic criterion in some population due to racial difference [14]. SD of HbA1c additionally predicted development of cardiovascular complication, independent of mean HbA1c [40].

How HbA1c variability affects the development of CAN is not clear, due to the paucity of research. However, we can find the answers from the association between HbA1c variability and retinopathy or nephropathy, because diabetic microvascular complications have similar etiologic characteristics based on the endothelial dysfunction [41]. First, the risk of microvascular complications tends to rise exponentially as HbA1c rises in previous studies [42]. In addition, greater HbA1c variability has been reported to be related to unhealthier lifestyles [16] and higher insulin resistance [43].

The chief strength of this study is its concurrent analysis of GV in CGM and HbA1c variability in the same study subjects, by using a relatively large CGM database. Although several studies have explored the association between GV and/or HbA1c variability and CAN, the number of subjects therein who underwent CGM was limited, or their HbA1c variability was not concurrently assessed [19, 20]. In addition, the CGM parameters of GV in our study included both relative and absolute parameters of GV. Finally, we used five cardiovascular reflex tests and Ewing’s definition of CAN, which have been commonly accepted in a number of previous studies. Some of those studies, which defined CAN as the presence of only one abnormal parasympathetic function test, could provide only limited estimates of CAN. Moreover, we applied age-specific reference values for interpreting results of parasympathetic tests. Use of single normative value for all ages will reduce the diagnostic discrimination of autonomic neuropathy tests and may result in false positive results in older patients [27].

There are a few limitations to this study. First, the study population of this study consisted of the small number of selected subjects with inadequately controlled diabetes requiring CGM. Because of the retrospective nature of the study prone to the selection bias, the results of this study should be cautiously extrapolated to other patient population. Second, CGM data was obtained for only three days, which may be too short of time to capture complete patterns of glycemia. Furthermore, all study subjects underwent CGM in outpatient clinic while they maintained usual unrestricted diet. Carbohydrate contents and meal composition can influence the short-term glycemic fluctuation. Third, there were various intervals among HbA1c measurements for each patient, although we adjusted the SD of HbA1c according to the number of measurements. Fourth, we are unable to confirm a direct causal relationship, in consequence of the limitations of a cross-sectional study.

In conclusion, CV in CGM and all parameters of HbA1c variability were independently associated with the presence of CAN in patients with inadequately controlled type 2 diabetes requiring CGM. HbA1c variability was also independently associated with the severity of the CAN. Although further prospective studies are required to determine the causal relationship, the results of this study indicate that the short-term relative GV and HbA1c variability might have a role in the pathophysiology of CAN in this patient population.

## Additional file

Additional file 1: Table S1. Reference values of the five autonomic function tests and the severity scores expressed as points. **Figure S1.** Scatter plots for the relationship between parameters of glycemic variability and total CAN score. SD: standard deviation; CV: coefficient of variance; MAGE: mean amplitude of glycemic excursions.

## Abbreviations

CAN: Cardiovascular autonomic neuropathy
GV: Glycemic variability
CGMS: Continuous glycemic monitoring system
SD: Standard deviation
CV: Coefficient of variance
MAGE: Mean amplitude of glycemic excursions
