# Supplementary material for: The association between glycemic variability and diabetic cardiovascular autonomic neuropathy in patients with type 2 diabetes
Source: Cardiovasc Diabetol. 2015 Jun 4;14:70. doi: 10.1186/s12933-015-0233-0 (PMC4462181; doi:10.1186/s12933-015-0233-0)
Supplement: Additional file 1: Table S1. — Reference values of the five autonomic function tests and the severity scores expressed as points. Figure S1. Scatter plots for the relationship between parameters of glycemic variability and total CAN score. SD: standard deviation; CV: coefficient of variance; MAGE: mean amplitude of glycemic excursions. [file 12933_2015_233_MOESM1_ESM.docx]

**Supplementary Table 1**. Reference values of the five autonomic function tests and the severity scores expressed as points.

| Autonomic function test | Measurement | Defined values | | |
| --- | --- | --- | --- | --- |
|  |  | Normal(0) | Borderline(0.5) | Abnormal(1) |
| ΔHR to deep breathing | E:I ratio | ≥ 1.06^†^ |  | < 1.06^†^ |
| Lying to standing ΔHR | 30 : 15 ratio | ≥ 1.07^‡^ |  | < 1.07^‡^ |
| Valsalva maneuver | Valsalva ratio | ≥ 1.12^#^ |  | < 1.12^#^ |
| Postural BP change | Fall in systolic BP | ≤ 10 | 11-29 | ≥ 30 |
| Sustained handgrip test | Rise in diastolic BP | ≥ 16 | 11-15 | ≤ 10 |

ΔHR, heart rate change; BP (mm Hg), blood pressure; E:I ratio, expiration to inspiration ratio.

^†^ Lower limit of the age-specific reference range of E:I ratio : age 20-24 years, 1.17; age 25-29, 1.15; age 30-34, 1.13; age 35-39, 1.12; age 40-44, 1.10; age 45-49, 1.08; age 50-54, 1.07; age 55-59, 1.06; age 60-64, 1.04; age 65-69, 1.03; and age 70-75, 1.02.

^‡^Lower limit of the age-specific reference range of 30:15 ratio : age 20-24 years 1.15; age 25-29, 1.14; age 30-34, 1.12; age 35-39, 1.11; age 40-44, 1.10; age 45-49, 1.09; age 50-54, 1.08; age 55-59, 1.07; age 60-64, 1.07; age 65-69, 1.06; and age 70-75, 1.06.

^#^Lower limit of the age-specific reference range of valsalva ratio : age 20-24 years, 1.43; age 25-29, 1.38; age 30-34, 1.33; age 35-39, 1.28; age 40-44, 1.24; age 45-49, 1.20; age 50-54, 1.16; age 55-59, 1.12; age 60-64, 1.08; age 65-69, 1.04; and age 70-75, 1.00.

**Supplementary Figure 1.** Scatter plots for the relationship between parameters of glycemic variability and total CAN score.

**(mg/dL)**

**(%)**

**(mg/dL)**

**(%)**

**(%)**
